# The Therapeutic Effect of Exercise on Anxiety and Bowel Oxidative Stress in the Maternal Separation Animal Model

**DOI:** 10.32598/bcn.9.10.450

**Published:** 2020-01-01

**Authors:** Ali Khorjahani, Maghsoud Peeri, Mohammad Ali Azarbayjani

**Affiliations:** 1. Department of Exercise Physiology, Central Tehran Branch, Islamic Azad University, Tehran, Iran.

**Keywords:** Maternal Separation (MS), Anxiety, Exercise, Brain, Gut, Oxidative stress

## Abstract

**Introduction:**

According to evidence, Early-Life Stress (ELS), mood disorders, and medical comorbidities, i.e. Irritable Bowel Syndrome (IBS), are correlated; however, the direct contribution of ELS to IBS manifestations is less understood. The current study aimed at evaluating the effect of voluntary exercise on the mitochondrial dysfunction of the bowel fibroblasts, following the confirmation of anxiety behavior.

**Methods:**

In this study, Postnatal Day (PND) rats underwent Maternal Separation (MS), as a valid animal model of the brain-gut axis dysfunction, in the days 2–14; three hours daily. On day 21, the study animals were divided into 4 groups, as follows: control, Running Wheel (RW) exercise, MS, and MS+RW groups. The study groups were housed in separate cages (4 rats per cage) until the onset of intervention. On day 60, the elevated plusmaze was used to assess anxiety-like behaviors; the level of oxidative stress biomarkers, i.e. Reactive Oxygen Species (ROS), Glutathione (GSH), as well as Adenosine Triphosphate (ATP) was measured to determine the gut mitochondrial function.

**Results:**

Findings revealed that ELS affected the gut energy metabolism in the studied rats; the negative effects of MS on anxiety and the gut mitochondrial dysfunction decreased via RW exercise during adolescence.

**Conclusion:**

Overall, anxiety behaviors and ROS production, leading to increased GSH and ATP levels, improved after RW exercise; this significantly impacts the function of colon secretory mitochondria. According to the positive effects of RW exercise on mitochondrial dysfunction in an ELS animal model, a potential relationship was found between the brain and gut in the study rats.

## Highlights

- Maternal separation is a valid animal model of brain-gut axis dysfunction in rats.
- Behavioral abnormalities were associated with mitochondrial dysfunction.
- The negative impact of maternal separation on the bowel mitochondrial function and animals’ behavior reduced following the voluntary exercise.

## Plain Language Summary

According to evidence, early-life stress, mood disorders, and some medical conditions, such as Irritable Bowel Syndrome (IBS), are correlated; IBS is among the commonest disabling diseases. This gastrointestinal illness is addressed by episodes of abdominal pain and bowel bloating. Unfortunately, the effect of antidepressant and anxiolytic agents, as well as other drug therapies on IBS treatment is unclear. Using a rat-model evaluation, current study findings indicated that the negative impacts of Maternal Separation (MS) on anxiety and the mitochondrial dysfunction of the gut could remarkably be controlled via Running Wheel (RW) exercise with no side effects.

## 1. Introduction:

Early-Life Stress (ELS) and stress-related psychiatric disorders are correlated with a disabling nature and a prevalent status (O’Mahony, Hyland, Dinan, & Cryan, 2011). Prior research indicated that mood disorders are accompanied by other comorbid conditions in the periphery organs, like the heart and gut (Barrett et al., 2012; Clarke, Cryan, Dinan, & Quigley, 2012; Eutamene et al., 2007; Klooker et al., 2009). Irritable Bowel Syndrome (IBS) is a common gastrointestinal illness addressed by the episodes of abdominal pain and bloating; in common sense, it is the brain-gut axis disorder (Zhao & Qian, 2014). This condition’s worldwide prevalence of 10%–15% is the most important reason for patients being visited by gastroenterologists. Furthermore, the prevalence of IBS is enhanced proportionately to Maternal Separation (MS) during childhood (Lowman, Drossman, Cramer, & McKee, 1987).

According to evidence, the nature and severity of symptoms, physiological complications, and psychiatric disorders significantly affect IBS treatment. However, the effect of antidepressant and anxiolytic agents, as well as other pharmacotherapies on IBS treatment, remains unclear (Ford, Talley, Schoenfeld, Quigley, & Moayyedi, 2008). Some studies have suggested physical exercise, as a novel and more efficacious method without adverse effects to concurrently treat IBS and anxiety (Carek et al., 2011; Herring et al., 2010).

Additionally, physical exercise increases the average life-span and improves health in humans (Radak, Chung, & Goto, 2008). Besides, other data have proven that exercise ameliorates the negative impact of MS stress on the behavior and incidence of oxidative stress in adult male rats (Radak, Taylor, Ohno, & Goto, 2001). An animal model study on male rats indicated that Running Wheel (RW) exercise could descend depressive-like behaviors, and reduce the expression of immune genes in the hippocampus (Sadeghi, Peeri, & Hosseini, 2016).

Evidence indicates that there are numerous mechanisms through which ELS induces its negative effects on the development of the brain and the formation of behavioral abnormalities (Herring et al., 2010). Studies indicated a correlation between oxidative stress and mitochondrial dysfunction and the pathophysiology of ELS-induced disorders (Taft & Keefer, 2016). Moreover, based on preclinical data, psychopathologies, such as depressive- and anxiety-like behaviors, are irritated following the experimental induction of colitis, indicating the bidirectional relationship between the gut and brain (Bercik et al., 2010). However, limited research explored the effects of ELS, exercise, and their interaction on the bowel mitochondrial function and anxiety-like behaviors. Therefore, the current study aimed at examining whether the rats with ELS induced by MS can develop anxiety-like behaviors. Besides, we investigated is the relationship between behavioral changes and the gut mitochondrial function. Finally, we assessed whether the ELS consequences on gut mitochondrial function are controlled by RW exercise. For this purpose, the gut fibroblasts, as an important component of the bowel for the isolation of mitochondria, were selected.

## 2. Methods

Twenty pregnant albinoes Wistar rats (gestation day 1) were obtained from the Pasteur Institute of Iran (Tehran, Iran) and housed under the standard laboratory conditions (22±2°C temperature, 50%±10% humidity, 12:12-h light-dark cycle, and standard rodents’ food and water ad libitum). All the study experiments were performed according to the National Institute of Health (NIH) Guide for the Care and Use of Laboratory Animals (NIH publication # 80–23), as well as the institutional guidelines for animal care and use (issued by the Department of Exercise Physiology, Central Tehran Branch, Islamic Azad University, Tehran, Iran).

Twenty pregnant rats were used in this study. Six male offspring were obtained for experiments and subsequently introduced to the MS paradigm based on the previously-published protocol (Amini-Khoei et al., 2017; Daniels, Marais, Stein, & Russell, 2012). The birthday was considered as the Postnatal Day 0 (PND 0), and then, within the PNDs 2 to 14, the pups experienced maternal separation every day for 180 minutes, beginning at 09:00 AM. On PND 14, they were returned to their mothers. Four male rats were assigned to each group in a cage until testing on PNDs 60–62. All study rats were healthy and with no signs of sickness or death during the experiments.

The study rats were equally assigned to 4 groups of 10-sample groups after the acclimation period. The groups were as follows: 1. The control (had no access to the RW); 2. RW group; 3. MS group; and 4. MS+RW treatment group. After the completion of the RW intervention on PND 60, behavioral tests were performed on the study rats (n=6–7 per group). Furthermore, on PND 60, the experimental group rats (n=3–4 per group) were beheaded under mild anesthesia, and the bowel was removed on an ice surface for biochemical assay.

On PND 21, RW exercise protocol was performed on the randomly selected rats (n= 10 per group). Accordingly, after a week of acclimation to the RW apparatus, MS rats underwent RW based on the Miladi-Gorji investigation protocol (Miladi-Gorji, Rashidy-Pour, & Fathollahi, 2012). Each exercise cage that housed a pair of MS rats was separated with perforated Plexiglas from the neighbor cage to avoid animals’ isolation and facilitate communication, and the RW apparatus was embedded in each cage. Similar conditions were provided for the control group, except for access to the RW. The Plexiglas wheels were 105 cm in circumference and 10 cm in width, with 5-g freely rotation (Novidan Tab, Iran). The rotation of the wheel was monitored hourly by a magnetic switch connected to and a counter placed outside the cage. There was no time limitation for using the RW apparatus, and the study rats were housed under the same conditions for 32 days until PND 60; their daily running distance was recorded in kilometers.

On RW exercise final day, behavioral tests were performed in rats (Pellow & File, 1986). The Elevated Plus Maze (EPM) relies on the natural fear of mice of open, unprotected, and elevated areas (Suo et al., 2013). The black Plexiglas-made EMP had two open (50×10 cm) and two closed (50×10×40 cm) cross-shared arms connected by a central platform (10 × 10 cm). The apparatus was located 60 cm from the ground with dim light. First, the study rats were placed in the central zone facing the open arm and provided with a 5-minute chance to explore the maze as described previously (Sadeghi et al., 2016). A video camera was embedded above the EMP to record the sessions; the amount of time spent on the open arms plus the open arm entries were scored. To eliminate residual odors of the previous rat, the maze was cleaned between the tests with 70% ethanol.

On the final day of RW exercise treatment, behavioral tests were performed in the study rats according to the protocol of Amini-Khoei et al., 2017. The passive behavior of rats (immobility time) was monitored, while they were forced to swim individually in a 50×20 cm (height × diameter) tall glass tank that 30 cm of it was water (23±1°C) to conduct the Forced Swim Test (FST). Immobility is referred to when the animal floats in the water and makes only those movements necessary to keep its head above the surface.

We considered mitochondrial isolation from bowel fibroblasts. After the completion of behavioral interventions and treatment with exercise, the study animals were kept at fasting state for 24 hours, then were anesthetized. After opening the intestinal cavity, the bowel was immediately removed and washed with Phosphate-Buffered Saline (PBS). The obtained tissue was quickly transferred to the DMEM medium, and after gently cutting, it was transferred to the Falcon Tube, and collagenase Intravenous (IV) (IV) was added to the medium (6–7 min). Then, the medium was filtered, and DMEM was re-added to the tube and placed instiller to isolate the bowel cells (10 min) uniformly. Then, the centrifuge was conducted at 400 RPM, and the supernatant was removed, and it was conducted again at 1000 g; the resulting sediment was suspended again in the DMEM medium. The resulting sediment contained bowel fibroblasts. In the second stage, the extraction of mitochondrial fractions from fibroblasts was performed on the centrifuge at 2500 g for 10 minutes; then the supernatant was collected into a Falcon tube and centrifuged at 8000 g (10 min), and the dark pallet tube containing the mitochondria was dispersed in terms of test in the desired buffer based on each test (Vicario et al., 2012; Wieckowski, Giorgi, Lebiedzinska, Duszynski, & Pinton, 2009). To ensure the purity of the obtained mitochondrial suspension, MTT assay was conducted to confirm the results based on previous reports (Shaki et al., 2012).

Adenosine Triphosphate (ATP) assay was performed by the ATP assay kit using colorimetric assay according to the manufacturer’s protocol (Abcam-83355). It is based on the phosphorylation of glycerol to generate a product that could be measured by Elisa-Reader at 570 nm based on a calibration standard curve.

The Dichloro-Dihydro-Fluorescein Diacetate (DCFHDA) assay was employed to measure the mitochondrial ROS production. Thus, 5 μL of 10 μM DCFH-DA was added to the supernatant, composed of respiratory buffer (Sonei et al., 2017). The amount of DCF, final fluorescent product, was determined at the excitation-emission wavelengths of 485 and 525 nm, respectively (Amiri et al., 2016; Sonei et al., 2017).

Glutathione (GSH) level was measured by a spectrophotometer (UV-1601 PC, Shimadzu, Japan) at 412 nm using Thermo Scientific Pierce Ellman’s Reagent (DTNB) following the emergence of yellow color based on calibration standard curve (Hosseini, Shaki, Ghazi-Khansari, & Pourahmad, 2014; Shaki et al., 2012).

All obtained data were expressed as Mean±SD. Oneway Analysis of Variance (ANOVA), followed by Tuckey’s posthoc test, was employed to make between-group comparisons. The level of significance was set at P<0.05.

## 3. Results

**Figure 1.**
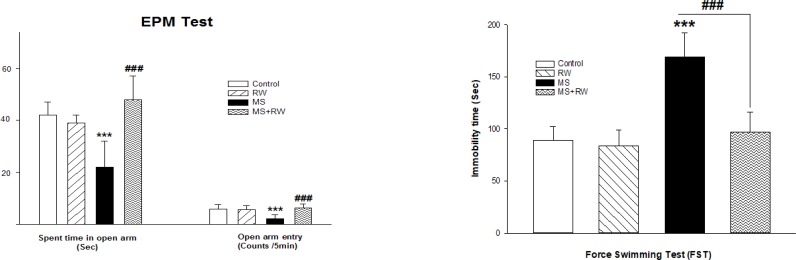
A. The effect of RW exercise on anxiogenic-like behaviors B. The effect of RW exercise on depressive-like behaviors The effect of chronic adolescent RW exercise on time spent on the open arm, open arm entry, and time spent in the center of EPM. Values are expressed as Mean±SD and were analyzed using one-way ANOVA followed by Tukey’s posttest test (n=7–8 in all groups). ^***^P<0.001 compared with the control group; ^###^P<0.001 compared with the MS groups. The effect of chronic adolescent RW exercise on immobility time on OFT. Values are expressed as Mean±SD and were analyzed using one-way ANOVA followed by Tukey’s posttest test (n=7–8 in all groups). ^***^P<0.001 compared with the control group; ^###^P<0.001 compared with the MS groups.

Anxiety-like behavior was assessed in the studied rats using the EPM test. In this test, the reduced frequency of the open arm entry and increased time spent in the closed arms indicate higher levels of anxiety. Using statistical analysis, it was determined that MS stress significantly causes anxiety-like behaviors. In other words, the number of entries to the open arm of apparatus in MS groups [F_(3,27)_=10.275, df=3, P<0.001, Figure 1] was lower than that of the control group. Moreover, the time spent in the open arm was decreased in the MS groups, compared to the controls [F_(3,27)_=17.930, df=3, P<0.001, Figure 1]. Besides, the achieved data revealed that the MS+RW group exhibited a significant increase in the open arm entry and the time spent in the open arms, compared to the MS groups, suggesting a significant reduction in the anxiety-like behaviors (Figure 1).

Increased immobility time in the FST was considered behavioral despair, i.e. a symptom of human depression (Amiri et al., 2016). One-way ANOVA results suggested a significant difference in the FST scores [F_(3, 27)_=38.462, P<0.001]. According to Figure 1B, the immobility time of MS rats was significantly higher than that of the controls (***P<0.001). Besides, the immobility time was shorter in the RW-treated MS rats, compared with the controls (^≠≠≠^P<0.001).

As per Table 1, ROS level increased time-dependently in the MS group, compared with the controls after 15 [F_(3,11)_=47.896, df=3, P<0.001] and 60 [F_(3,11)_=32.641, df=3, P<0.001] minutes. This finding indicates the involvement of oxidative stress in isolated mitochondria obtained from bowel fibroblasts. The ROS production rate had no significant elevation in the MS+RW rats after a 60-minute exposure, compared to the controls (P>0.05).

**Table 1.** The Effect of RW exercises on ROS formation

**Groups**	**ROS Formation (%)**	

	**15 min**	**60 min**
Control	4±2	13±4
RW	4±2	8±3
Maternal Separation (MS)	32±6	50±9
MS+RW	5±2	19±5

**Figure 2.**
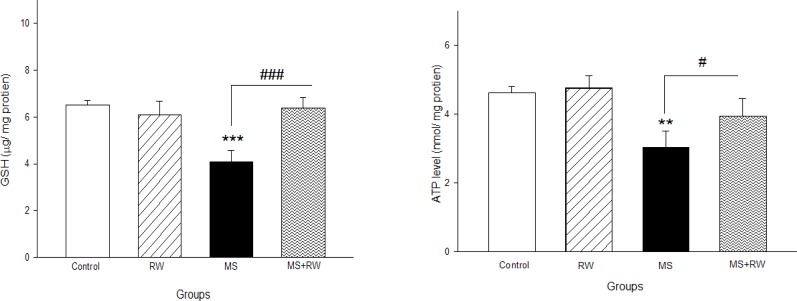
The effect of RW exercises on GSH level Values are expressed as Mean±SD. ^***^P<0.001 compared with the control group and ^###^P<0.001 compared with the MS group.

A significant decrease was observed in the GSH level of the MS rats, in comparison with the controls (P<0.05). However, in the RW exercise groups, the GSH level had a significant elevation, compared to the MS group [F_(3,11)_=22.941, df=3, P<0.001] (Figure 2).

**Figure 3.**
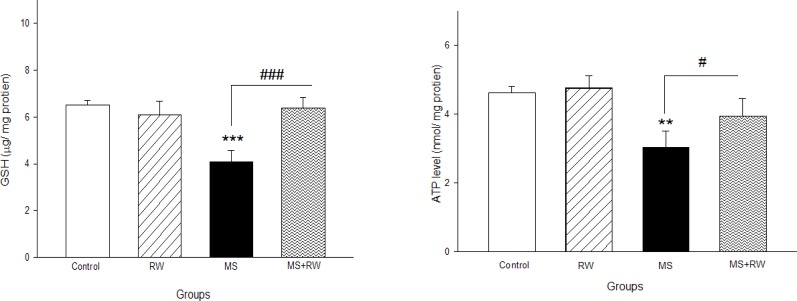
The effect of MS in childhood on mitochondrial ATP level Values are expressed as mean±SD. ^*^P<0.05; ^**^P<0.01; ^***^P<0.001compared with the control group and ^#^ P<0.05; ^##^ P<0.01; ^###^ P<0.001 compared with the MS group.

The ATP level significantly decreased in the MS rats, compared to the controls [F_(3,11)_=13.5, df=3, P<0.001]. However, the RW exercise groups demonstrated a significant increase in ATP level, compared to the MS groups (Figure 3).

Reactive oxygen species were measured by fluorescent dye DCFH-DA. The change in fluorescence was determined at 485 nm for excitation and 525 nm for emission after 15 and 60 min incubation, using fluorometry.

## 4. Discussion

MS stress in rodents is a non-pharmacological method that could induce behavioral changes, such as depression, anxiety, and inducing visceral hypersensitivity to rectal distention (Carek et al., 2011; Sadeghi et al., 2016). The current study findings supported experiencing MS-induced anxiety- and depressive-like behaviors and mitochondrial dysfunction in bowel fibroblasts. The obtained data indicated that oxidative stress and antioxidant imbalance contribute to the negative effects of ELS on gut energy metabolism. Besides, RW exercise could reverse the ELS-induced anxiogenic behavior and abnormalities in the gut mitochondrial function.

Several lines of clinical and preclinical research have suggested that early life stress and trauma could alter GI motility, disrupt intestinal epithelial barrier, and activate gut mucosal inflammatory responses during adulthood (Cryan & Holmes, 2005; Nicholl et al., 2008; O’Mahony et al., 2009; O’Malley, Julio-Pieper, Gibney, Dinan, & Cryan, 2010). According to clinical data, 50%–60% of patients with IBS also have psychiatric disorders, like depression and anxiety (Vicario et al., 2012). According to the current study, the MS-treated group presented anxiety-like behaviors. Such behaviors were characterized by spending a shorter time on the open arms and less frequency of the open arms in the EPM. Besides, the achieved data suggested that RW exercise ameliorated anxiety-like behaviors in the study rats.

Vicario et al. argued that the reversible mitochondrial damage and upregulated corticotropin-releasing factor receptor type-1 are induced in the gut following the incidence of chronic social stress; they could cause IBS-like gut dysfunction (Vicario et al., 2012). Additionally, physical activity and exercise induce widespread neurobiological adaptations and could reduce anxiety-like behaviors, enhance neurogenesis via promoting IGF1 and BDNF activity (Droste et al., 2003; Schoenfeld, Rada, Pieruzzini, Hsueh, & Gould, 2013), and reduce ACTH hormones and corticosterone levels (Hare, Beierle, Toufexis, Hammack, & Falls, 2014; Wang, Chen, Lin, Jhong, & Chen, 2014). A study reported the expression of the NR2A subunit of NMDA receptors increased following MS in the hippocampus, i.e. crucial in the incidence of depression- and anxiety-like behaviors. Moreover, the negative impacts of MS on behavior and mitochondrial function was controlled via RW in adult rats (Fattahi, Peeri, Azarbayjani, & Hosseini, 2017). Therefore, MS could enhance glutamatergic signaling, which causes anxiety-like behaviors and mitochondrial dysfunction in bowel, similar to depressive-like behaviors.

Accordingly, evidence indicated that dysfunction in the mitochondrial bioenergetics influences the pathophysiology of psychiatric disorders; i.e. depression and anxiety via different routes, such as the activation of oxidative stress or inflammatory pathways, the activation of NMDA receptor, and altering the plasticity of neurons (Brown & Bal-Price, 2003; Gardner & Boles, 2011; Klinedinst & Regenold, 2015; Morava & Kozicz, 2013). A study revealed the induction of mitochondrial dysfunction and oxidative stress in the bowel fibroblasts of MS adult rats (Vicario et al., 2012). The results of ROS production demonstrated that the rate of ROS production was higher in MS rats, compared with the controls in the mitochondria obtained from bowel; therefore, ROS overproduction in mitochondria is induced following the early-life maternal separation, i.e. associated with mitochondria dysfunction and oxidative stress in the bowel cells (Table 1).

In line with these results, the findings of a study indicated that MS exposure caused a significant decrease in GSH level, which supports increased ROS production. Furthermore, another study suggested that MS negatively impacted GSH level, as the main antioxidant system, and adversely affects mitochondrial permeability transition pore opening. ATP levels in MS rats were decreased compared to the control groups. This finding indicates that early life can disrupt the metabolism of energy in the bowel mitochondria isolated from fibroblast in adult rats.

The present investigation revealed the significant reduction of ROS production on the isolated mitochondria obtained from bowel tissue following treatment with RW exercise (Bachur et al., 2007; Salim et al., 2010). Improving the antioxidant system following RW exercise and decreasing superoxide radical production or ROS production have a protective role against MS-induced changes. Besides, the study findings were consistent with those of other studies indicating exercise increased GSH level (Bachur et al., 2007; Hovanloo, Hedayati, Ebrahimi, & Abednazari, 2011; Oh-Ishi et al., 1994; Rezin et al., 2009; Sonei et al., 2017). The inconsistency between the results is due to the difference in the antioxidant and oxidant scavenger pattern or difference in the intensity and duration of exercises. In terms of recommendations, finding indicated the induction of mitochondrial dysfunction in the bowel fibroblast by ELS-derived stress; thus, the impact of exercise on anxiety might be affected by the intensity and duration of exercise, resting time after exercise and the severity of the psychiatric disorder.

## 5. Conclusion

the current study results suggested that experiencing stress due to early-life maternal separation is a risk factor for medical comorbidities; i.e. bowel complications in adulthood; however, t RW exercise during adolescence indicated anxiolytic and protective effects on the negative outcomes of ELS on the mitochondrial function of the bowel.
